# Supplementary material for: Unveiling the Protein Components of the Secretory-Venom Gland and Venom of the Scorpion Centruroides possanii (Buthidae) through Omic Technologies
Source: Toxins (Basel). 2023 Aug 9;15(8):498. doi: 10.3390/toxins15080498 (PMC10467079; doi:10.3390/toxins15080498)
Supplement: Supplementary file 1 [file toxins-15-00498-s001.zip › Supplementary Table S3.pdf]

Supplementary Materials: Unveiling the Protein Components of the Secretory-Venom Gland and Venom of the Scorpion Centruroides possanii (Buthidae) through Omic Technologies

Patricia Elizabeth GarcíaVillalvazo, Juana María Jiménez-Vargas, Gisela Jareth Lino-López, Erika P. Meneses, Manuel J. Bermúdez-Guzmán, Carlos Eduardo Barajas-Saucedo, Iván Delgado Enciso, Lourival D. Possani and Laura Leticia Valdez-Velazquez

Table S3. Molecular weights (MW) of the venom components from C. possani. A total of 180 MW was found. A dimer of 7274.18 molecular mass is shown in red text. The values < 3500 Da are monoisotopic masses, and >3500 Da are average masses.

RT (min)	MW (Da)	RT (min)	MW (Da)
0-30	1357.70, 1438.57, 2138.03, 2411.09, 2720.08, 3907.76, 4044.71,4269.72	90-100	814.46, 840.46, 845.42, 852.40, 862.38, 876.46, 942.54, 1198.60, 1486.92, 1573.76, 1870.06, 1975.18, 2768.07, 2841.36, 3294.71, 3420.31, 3955.82, 4018.24, 4037.81, 4113.98, 4224.01, 4237.96, 4306.01, 4784.12, 4828.90, 5596.57, 6589.94, 6907.16, 6946.17, 6963.15, 7116.16, 7126.15, 7165.31, 7338.20, 7576.64, 7675.36, 7745.50, 7847.63
30-40	1318.62, 1415.70, 1446.54, 1594.76, 1756.80, 2557.18, 2719.08, 2826.40, 3246.90, 3827.70, 3875.69, 4022.53, 4070.80, 7353.24	100-110	1530.80, 1681.77, 1967.16, 2426.39, 2788.67, 3479.55, 3583.66, 3810.88, 4047.71, 4072.77, 4111.96, 4240.98, 5162.38, 5588.57, 6195.64, 6332.62, 6350.81, 6385.34, 6464.85, 6972.05, 7109.98, 7153.12, 7228.12, 7237.18, 7247.76, 7577.37, 7971.24, 8244.82
40-50	3819.69, 5384.17, 5559.89, 5902.71, 6469.78, 7145.05, 7743.53, 9150.13	100-120	1167.74, 1547.49, 2927.75, 3128.64, 6088.96, 6217.12, 6602.10, 6971.12, 7143.40, 7239.77, 7264.30, 7268.31, 7274.18, 7332.16, 7372.13, 7466.33, 7468.72, 7841.47, 14550.39
50-70	966.58, 1019.59, 1030.44, 1163.75, 1348.73, 1670.93,	120-130	1735.76, 1855.80, 1877.80, 2323.35, 2435.45, 2747.70,

	2112.02, 2134.00, 2192.91, 2483.24, 2516.04, 2527.03, 2541.03, 2837.31, 3418.29, 3894.74, 3952.80, 4014.66, 4035.77, 4109.95, 4225.83, 4303.04, 4506.43, 4825.87		3629.05, 3651.63, 3779.39, 5386.45, 5932.50, 6470.74, 7048.99, 7231.12, 7380.15, 7551.39, 7579.44, 7746.54, 9094.08, 9144.17
70-90	1060.49, 1417.70, 1489.79, 4235.72, 6397.75	130-150	1607.72, 1971.31, 2185.12, 5564.36, 5678.44, 6197.83, 6689.20, 7277.17, 7399.38, 7462.04, 7637.75, 7748.60, 9180.07, 9550.00, 10880.16, 16096.96
