# Supplementary figures and images for: Unveiling the Protein Components of the Secretory-Venom Gland and Venom of the Scorpion Centruroides possanii (Buthidae) through Omic Technologies
Source: Toxins (Basel). 2023 Aug 9;15(8):498. doi: 10.3390/toxins15080498 (PMC10467079; doi:10.3390/toxins15080498)

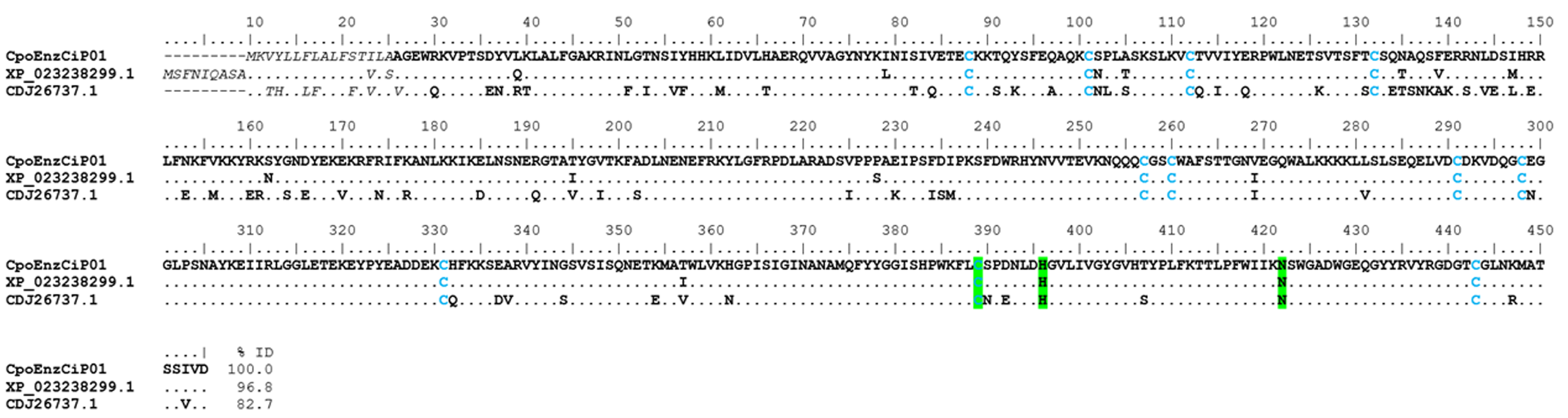

Supplement: Supplementary file 1 [file toxins-15-00498-s001.zip › Supplementary Figure S1.tif]

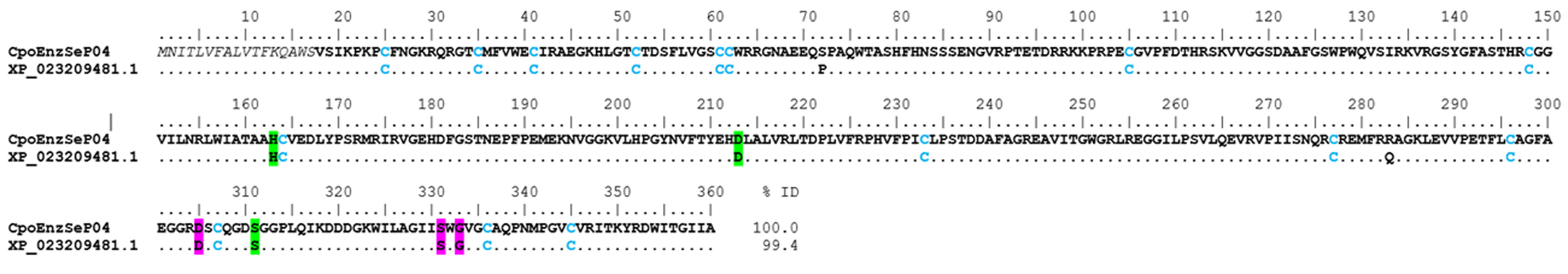

Supplement: Supplementary file 1 [file toxins-15-00498-s001.zip › Supplementary Figure S2.tif]

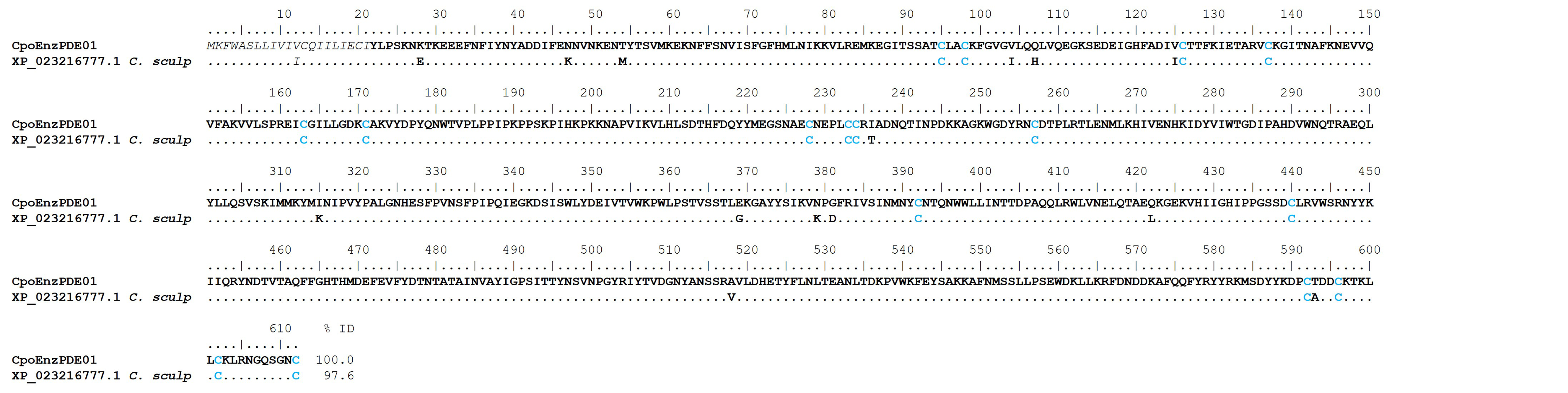

Supplement: Supplementary file 1 [file toxins-15-00498-s001.zip › Supplementary Figure S3.tif]
